# Trends of Antibiotic Resistance in Mesophilic and Psychrotrophic Bacterial Populations during Cold Storage of Raw Milk

**DOI:** 10.5402/2012/918208

**Published:** 2012-03-12

**Authors:** Patricia Munsch-Alatossava, Jean-Pierre Gauchi, Bhawani Chamlagain, Tapani Alatossava

**Affiliations:** ^1^Division of Food Technology, Department of Food and Environmental Sciences, University of Helsinki, 00014 Helsinki, Finland; ^2^Unité de Mathématiques et Informatique Appliquées (UR 341), Centre de Jouy en Josas, Institut National de la Recherche Agronomique, Domaine de Vilvert, 78352 Jouy en Josas, France

## Abstract

Psychrotrophic bacteria in raw milk are most well known for their spoilage potential and cause significant economic losses in the dairy industry. Despite their ability to produce several exoenzyme types at low temperatures, psychrotrophs that dominate the microflora at the time of spoilage are generally considered benign bacteria. It was recently reported that raw milk-spoiling Gram-negative-psychrotrophs frequently carried antibiotic resistance (AR) features. The present study evaluated AR to four antibiotics (ABs) (gentamicin, ceftazidime, levofloxacin, and trimethoprim-sulfamethoxazole) in mesophilic and psychrotrophic bacterial populations recovered from 18 raw milk samples, after four days storage at 4°C or 6°C. Robust analysis of variance and non parametric statistics (e.g., REGW and NPS) revealed that AR prevalence among psychrotrophs, for milk samples stored at 4°C, often equalled the initial levels and equalled or increased during the cold storage at 6°C, depending on the AB. The study performed at 4°C with an intermediate sampling point at day 2 suggested that (1) different psychrotrophic communities with varying AR levels dominate over time and (2) that AR (determined from relative amounts) was most prevalent, transiently, after 2-day storage in psychrotrophic or mesophilic populations, most importantly at a stage where total counts were below or around 10^5^ CFU/mL, at levels at which the milk is acceptable for industrial dairy industrial processes.

## 1. Introduction

In developed countries, the sanitation of raw milk is monitored by “total” bacterial counts or SPC (standard plate count). The standard for Grade A or 1 raw milk is an SPC value less than 1.0 × 10^5 ^CFU/mL [1]. Cooling of raw milk to less than 6°C (typically 3 to 4°C in the farm tank following milking), followed by storage at temperatures below 6°C during transportation to the dairy plant, ensures the quality of raw milk. Because milk is a highly suitable growth medium, many bacterial genera have adapted to this cold-temperature environment by producing exoenzymes (such as proteases or lipases) that can withstand the typical heat treatments milk is subjected to, and they consequently cause significant economic loss to the dairy industry [2–5]. Psychrotrophic bacteria (able to grow at temperatures ranging from 0°C to 7°C) [6] are well known for their ability to degrade both raw and processed milk components which may explain why raw milk psychrotrophs are mainly considered for their spoilage features, with some exceptions like the human pathogens* Bacillus cereus* (toxin-producing strains) [7], or *Listeria monocytogenes *[8]. Most genera in psychrotrophic communities are Gram-negative [6] and are generally considered to be benign bacteria. Recently, Gram-negative bacteria have come under higher scrutiny worldwide because many genera host species considered as human opportunistic pathogens, which may have antibiotic multiresistant-traits, and infections due to those species may eventually be untreatable [9, 10].

Antibiotic resistance (AR) is a consequence of antibiotic (AB) use, overuse, and misuse; AB administered to animals used for food products is an important source of antimicrobial-resistant bacteria that can spread to humans through the food supply [11–14]. The distribution spectrum and magnitude of the AR gene pool in food-borne pathogens is alarming. Recent studies have concluded that large AR gene pools are present in commensal bacteria in many ready-to-eat products and implicated food production and processing environments in the evolution and dissemination of AR [15, 16]. The demonstration of the presence of transmissible AR genes in the human food chain was earlier made [17]; thus, food can be considered to be a direct source of AR genes that is consumed daily.

Many studies have determined the prevalence of AR among mastitis pathogens [18, 19], several compared the AR of conventional and organic dairy farming practices [20, 21], and others have determined the prevalence of AR among Gram-negative enteric bacteria in bulk milk tanks [22], or mastitis milk samples [23]. However, little is known regarding the AR of raw milk-spoiling psychrotrophs. A phenotypic-based study indicated representatives of *Pseudomonas, Acinetobacter,* and *Stenotrophomonas* [24], and the 16SrDNA gene sequences confirmed the identity of some isolates categorized in more risky genera (unpublished data). We investigated the AR of some isolates and observed that multiple AR was rather common, and its incidence increased during the cold chain of raw milk storage and transportation: moreover, a constant increase in AR for isolates originating from farms, lorries, or silos was observed for the following ABs: ticarcillin, aztreonam, cefepime, ofloxacin, and trimethoprim-sulfamethoxazole; bacterial isolates from milk stored in silos more frequently exhibited resistance to clavulonic ticarcillin-clavulAnic acid, ceftazidim, imipenem, colistin, gentamicin, and ofloxacin compared with farm isolates; unfortunately this study considered independent samples [25]. To further investigate whether the cold storage of raw milk promotes an increase in AR traits, we evaluated the AR of total mesophilic and psychrotrophic populations of raw milk stored at 4 or 6°C as function of time and examined the effects of temperature and time upon the AR. To account for low initial bacterial counts (a typical characteristic of high quality farm milk) and variations between milk from different farms more likely to be reflecting individual farm management practices, we studied commingled milk samples withdrawn from lorry tanks that constitute somehow an intermediate stage of the cold chain of raw milk storage and transportation.

## 2. Materials and Methods

### 2.1. Cold Storage of Raw Milk Samples

All raw bovine milk samples were obtained from lorry tanks of the Valio Ltd dairy company (Helsinki, Finland). Representative samples of the lorry contents were collected in sterile bottles, by licensed milk haulers shortly after the lorries arrived at the dairy. The samples were then kept on ice until arrival at Helsinki University, at which time 100 mL aliquots were added to sterile 250 mL bottles. Six bottles were placed on a multiplace magnetic stirrer (Variomag) and partially immersed in a refrigerated water bath (MGW Lauda MS/2), which allowed a constant temperature (monitored by an immersion thermostat) to be maintained (modified from [26]). The raw milk was continuously mixed at 220 rpm and kept at 4 ± 0.1°C or 6 ± 0.1°C for 4 days.

### 2.2. Enumeration of Bacterial Populations

Microbiological analyses were performed immediately after milk samples arrived. All bacterial counts were determined from triplicate or quadruplicate agar cultures at days 0 (shortly after receiving the samples) and 4 for experiments I (4°C) and II (6°C), and at days 2 and 4 for experiment III (4°C), (Table 1) by aseptically removing 500 *μ*L of raw milk, serially diluting the sample in a saline solution (0.85% NaCl), and spreading 50 *μ*L on Mueller-Hinton (LabM) agar. Four antimicrobial agents (gentamicin (G), ceftazidim (C), levofloxacin (L), and trimethoprim-sulfamethoxazole (TS at a ratio of 1/19) (Sigma)) were prepared according to the EUCAST guidelines [27]. The AB solutions were freshly prepared by dissolving powders in the following solvents: water for G, 0.1 M phosphate buffer (pH 7) for C, 0.1 M NaOH for L, 0.1 M lactic acid for T, and 95% ethanol for S (EUCAST 2000). With the exception of S, all AB solutions were filter sterilized, prior to the addition to adequately cooled agar. The AB concentrations were 16 mg/L for GI, 4 mg/L for GII, 32 mg/L for CI, 8 mg/L for CII, 8 mg/L for LI, 2 mg/L for LII, 8 mg/L trimethoprim with 152 mg/L sulfomethoxazole for TSI, and 4 mg/L trimethoprim with 76 mg/L sulfomethoxazole for TSII, which correspond to the MICs (GII, CII, LII, and TSII) to 4-fold the MIC (GI, CI, LI), or 2-fold the MIC (TSI) as indicated by EUCAST for pseudomonads. Agar plates were stored overnight at 4°C and protected from light. Following initial analysis, the plates were incubated for 2-3 days at 30°C or for 10 days at 7°C to enumerate the “total” bacteria (mainly mesophiles) and psychrotrophs, respectively.

### 2.3. Statistical Methods

 Two outputs were considered to quantify AR. The first was AR prevalence, which is generally defined as the percentage of resistant bacteria considering the corresponding “total” bacteria enumerated in the same conditions, and the second was Rapd which was used to overcome perturbations due to excessive low versus high bacterial counts and variation among plates. Rapd is defined for a particular condition X and characterized by a combination of factors including population type (psychrotrophs (P) or mesophiles (M)), sampling day (d = 0, 2 or 4), storage temperature, AB type, and AB concentration. Rapd corresponds to the number of bacterial colonies (CFU/mL) in a certain condition (determined by a combination of the previous listed factors) divided by the total number of bacteria on the corresponding control plates (in the absence of the AB). To overcome variation between raw milk samples, which was mainly due to the initial microflora, statistical analyses were based on the rank of the Rapd instead of the Rapd itself. The use of the ranking enabled a more robust quantification of the AR regardless of the variation among the four considered factors (milk storage temperature and time, as well as AB type and concentration). The reliability of the results was determined by robust ANOVA and nonparametric statistics which are suited for analyses of heterogeneous data such as microbiological data.

The following statistical methods, previously applied [28], were also used for data analysis. (1) In a view of a simple description of data, usual statistical tools (USTs) for mean, standard deviation, median, and box-and-whiskers-plots were generally used to represent bacterial enumeration (expressed as CFU/mL or in decimal log-units). (2) In a view to improve the robustness of the conclusions on ranked data, analysis of variance (ANOVA) on Rapd ranks as a function of the studied factors (milk storage temperature and time as well as AB type and concentration) was used to detect significantly influential factors (main effects or interaction effects). In the ANOVA framework, it is common to make multiple comparisons of means. Here, the Ryan-Einot-Gabriel-Welsch (REGW) test for multiple comparisons of ratio means was used. (3) Nonparametric statistics (NPSs), including Wilcoxon, Kruskal-Wallis, median two-sample, and Van der Waerden tests, were used [29]. These analyses were performed using the NPAR1WAY procedure of the SAS/STAT statistical software (version 8.1) of the SAS Institute (NC, USA). The objective of these tests was to confirm or infirm statistically significant change of the Rap4 versus Rap0 means (Rapd, defined for one condition considering a combination of factors set at a particular level). For UST, the AR percentages were used to compare the number of mesophiles (or “total counts”) with psychrotrophs, respectively. However, Rapd was used for ANOVA and NPS analyses.

## 3. Results and Discussion

### 3.1. Raw Milk Samples

The microbiological quality of the 18 analyzed raw milk samples was excellent: 17 of 18 samples had initial bacterial counts less than 5.0 × 10^4^ CFU/mL (4.7 in decimal log-units) (Table 1). At day 0, the psychrotrophs/“total counts” ratios were lower than 0.9 in eight samples and higher than 0.9 in ten samples (Table 1). No correlation between the initial “total” bacterial load and the fraction of psychrotrophs present in raw milk samples was detected. Assuming that a higher ratio value corresponds to milk that spent more time in cold storage conditions, we estimate that the raw milk samples were subjected to “different storage times” prior to the analyses.

### 3.2. AR Prevalence among Mesophilic versus Psychrotrophic Bacteria at the Start of the Analyses (Day 0) Evaluated for Each AB and Bacterial Community Type

#### 3.2.1. Descriptive Statistics

**Figure 1:**
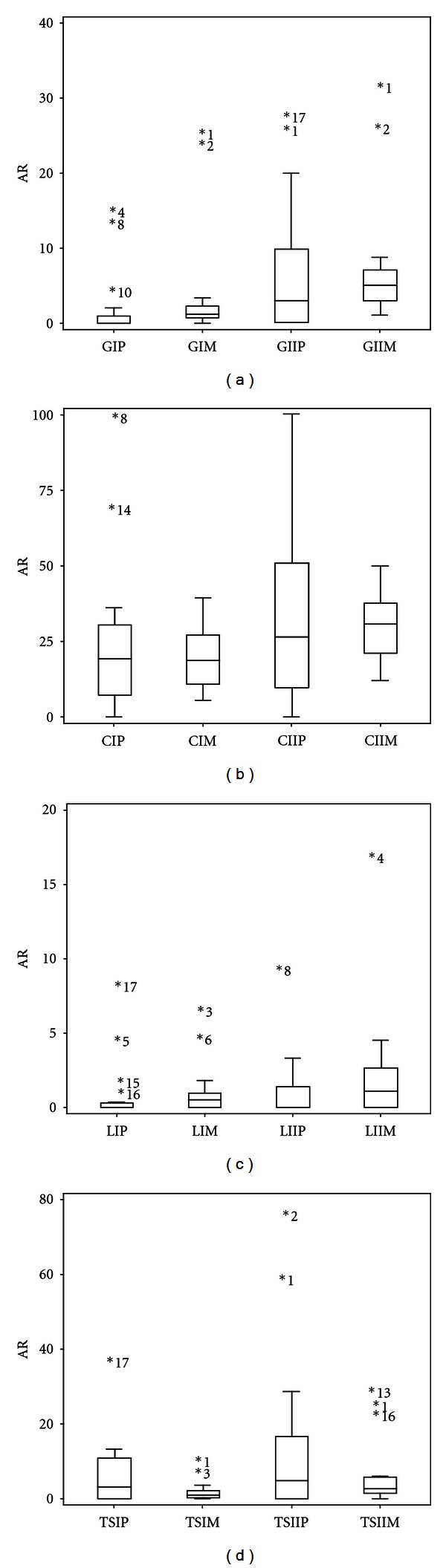
Box- and-whiskers plots of the initial AR percentages distributions. The AR values correspond to gentamicin (G, 1a), ceftazidim (C, 1b), levofloxacin (L, 1c), and trimethoprim-sulfamethoxazole (TS, 1d) for psychrotrophic (P) and mesophilic (M) bacterial populations (I = 4 × MIC for G, C, L and 2 × MIC for TS; II = MIC). The inner box lines represent the geometric medians, while the outer box lines represent the 25th and 75th data percentiles; the outliers are indicated beside their sample number. Sample 8 that showed an AR of 100% for GIIP is not included in Figure 1(a).

**Figure 2:**
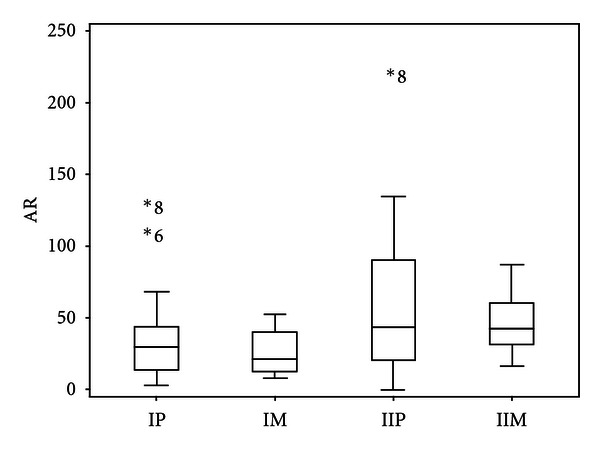
Box- and-whiskers plots of the distribution of the total initial AR percentages for psychrotrophs (P) and mesophiles (M). The AR values correspond to the two AB concentrations (I = 4 × MIC for G, C, L, and 2 × MIC for TS; II = MIC). Outliers are indicated under their sample number.

The AR for each AB and population type is summarized in Figures 1 and 2. For the majority of the samples analyzed shortly after reception, higher AR percentages with larger box boundaries were observed with lower AB concentrations regardless of the AB type, which is indicative of an AB-concentration-dependent response (Figures 1 and 2). With some exceptions, mesophilic populations (M) had AR percentages with gentamycin (G) of 0%–3% (GIM) and 1.1%–9% (GIIM) (Figure 1(a)). The psychrotrophic populations (P) were more heterogeneous and exhibited slightly lower resistance to GIP (0%–2%) than GIIP (0%–30%). The AR prevalence to ceftazidim was higher than for the other tested ABs and was highest for the psychrotrophic populations on CII (Figure 1(b)); although the median values of CIP and CIM, and of CIIP and CIIM, showed only minor differences, the psychrotrophic populations had more heterogeneity in AR prevalence, especially for the lower ceftazidim concentration (CII). Approximately one-fourth of the samples had AR percentages of 50%–100%, and 38%–50% for CIIP and CIIM, respectively. AR to levofloxacin (L, Figure 1(c)) was the least prevalent among the considered antibiotics at both concentrations but was moderately higher for mesophiles than for psychrotrophs. The prevalence of AR to trimethoprim-sulfamethoxazole (TS, Figure 1(d)) was higher for the psychrotrophs (P) than mesophiles (M) as indicated by the median values and boundaries of the box plots. The heterogeneity among samples was also highest for psychrotrophs.

The percentages of AR observed with high AB concentrations were less than those observed at low AB concentrations for all mesophilic populations and for 13 of 18 psychrotrophic populations. AB-concentration-dependent responses were apparent when considering the boundaries of the box-and-whiskers plots depicting the total AR percentages, comparing IM and IIM with IP and IIP (mesophiles (M) or psychrotrophs (P), present on plates with high (I) or low (II) antibiotic concentrations) (Figure 2). The median value observed for the psychrotrophs (IP) exceeded by 10% the value of the mesophiles (IM) with the highest AB concentrations; the 75th percentiles had AR maximums of 70% (IP) and 52% (IM); the top quartiles were comprised between 45% and 70%, 40%, and 50% for the psychrotrophs (IP) and mesophiles (IM), respectively. The distribution of AR in the mesophiles was more homogeneous than in the psychrotrophs: half of the samples had total resistance percentages ranging from 50% through 100% (IM) and 30% through 130% (IP). Although the median value was slightly greater than 40% for both types of populations, with the low antibiotic concentrations half of the samples had total AR of 20% to 90% (IIP) and 30% to 60% (IIM) (Figure 2). The 75th interquartile had AR of 90%–135% (psychrotrophs) and 60%–90% (mesophiles), respectively. The total percentages of AR (evaluated using four ABs, regardless of concentration) never exceeded 100% for the mesophilic populations, contrarily to psychrotrophic populations present in four milk samples. This result could be indicative of a higher prevalence of multiresistant bacteria among psychrotrophs than mesophiles.

A link between “total initial counts”, the psychrotrophs/“total counts” ratios, and the number of encountered AR fractions among psychrotrophs versus mesophiles in a given raw milk sample is not apparent. For example, sample 8 had low initial counts and a low psychrotrophs/mesophiles ratio but revealed the highest AR for the psychrotrophs, which greatly exceeded the AR of the corresponding mesophiles.

The AR was less dependent on the “age of the raw milk” (the time that elapsed between milking and the time of analyses which could be somehow estimated from ratios of psychrotrophs/mesophiles) but rather more dependent on the initial bacterial microflora.

#### 3.2.2. ANOVA Results

The mean values of the Rap0 ratios determined at day 0 were 0.2396 and 0.3448 (C), 0.0544 and 0.0676 (G), 0.0485 and 0.0939 (TS), and 0.0171 and 0.0086 (L) for the mesophiles and psychrotrophs, respectively. Regardless of the bacterial population type, AR was most prevalent for C and least for L. With the exception of L, all mean Rap0 values analyzed with the REGW test for psychrotrophs were superior to those observed for mesophiles which confirmed previous observations (Section 2.1). ANOVA performed on Rap0 and on Rap0 rank (see Rap0 definition in section 2) revealed significant main effects for the mesophiles. *F* values (from the Fisher-Snedecor test, *P* < 0.0001) were 82.73, 176.95, and 40.27 for storage temperature, AB type, and AB concentration, respectively. The effects due to AB type and the interaction between storage temperature and AB type were partially sample dependent. The REGW range test for Rap0 considered the four ABs distinct, but the effects of L and TS were similar. To overcome perturbations due to sample 1, which was considered different (highest Rap0 mean value), a second REGW range test was performed for Rap0 after eliminating the sample 1; the test confirmed the importance of the considered factors. The *F* values (from the Fisher-Snedecor test, *P* < 0.0001) were 20.22, 108.96, and 20.14 for storage temperature, AB type, and concentration, respectively, for samples 2–6. Both approaches confirmed the ranking of the ABs according to their decreasing prevalence: C, G, TS, and L.

The AR for psychrotrophs was more sample dependent (*F* value of 18.35, *P* < 0.0001). A significant difference was observed for the four ABs (*F* value of 68.84, *P* < 0.0001), and significant interactions between storage temperature and sample (*F* value of 15.31) were detected. Unlike the mesophiles, the encountered variability relied on a high sample/temperature interaction. The REGW multiple range test ranked C and L in the 1st and 4th positions, respectively, in AR prevalence for both mesophiles and psychrotrophs. G and TS were ranked in 2nd and 3rd position, respectively, for the mesophiles; the rankings were reversed for the psychrotrophs where the AR to TS was the second most prevalent. The analyses considered the four ABs distinct, although TS and L induced similar AR levels for the mesophiles, whereas TS and G induced similar AR levels for the psychrotrophs.

#### 3.2.3. Evolution of the AR of Psychrotrophic Populations from Two Raw Milk Samples (13 and 16, Table 1) Stored at 4°C for 4 Days

**Figure 3:**
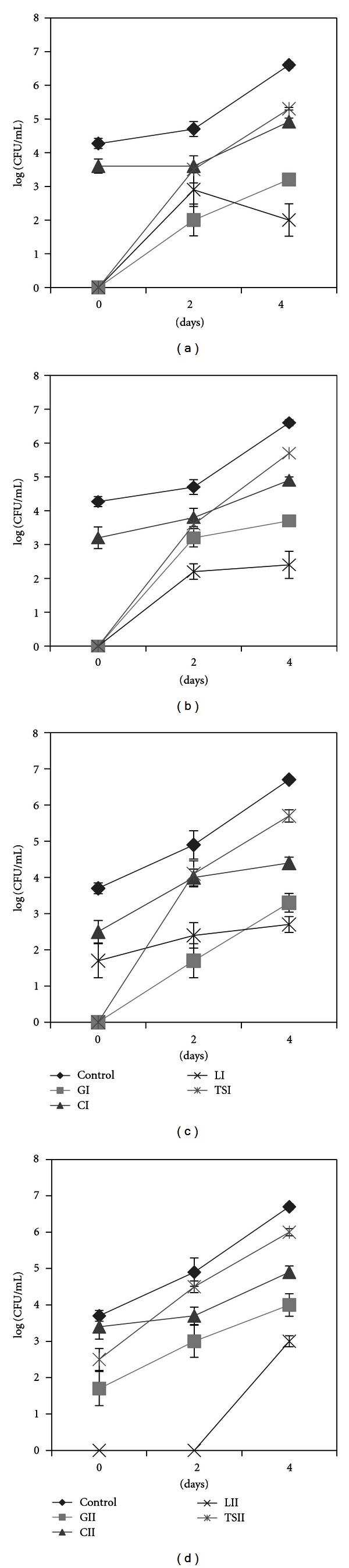
AR trend of psychrotrophs in raw milk samples 13 (a and b) and 16 (c and d).

Figure 3(a) depicts the trend of ceftazidim-resistant psychrotrophs (CI) which nearly parallels the total psychrotrophic population in raw milk sample (13); the differences were approximately 0.7, 1, and up to 2 log-units at days 0, 2, and 4, respectively. While no resistant isolates were detected on day 0 on trimethoprim-sulfamethoxazole (TSI) plates, the AR reached the level of C-resistant isolates (about 3.5 log-units) at day 2. The growth kinetics between days 2 and 4 were almost undistinguishable between isolates recovered from CI and TSI plates. A similar trend (when compared to TSI) was observed for gentamicin (GI), where the counts were approximately 2 and 3 log-units at days 2 and 4, respectively, which followed the trend of the control but were 2.5 and 3 log-units lower than the counts on control plates. Although not detected at day 0, levofloxacin-resistant (LI) isolates were present on day 2 at a level of 3 log-units but declined to approximately 2 log-units at day 4. No differences were observed between isolates grown on CII and TSII plates, compared to the trends observed for CI and TSI, respectively (Figure 3(b)). The AR levels detected on GII and LII were in the same range (approximately 2-3 log-units) but inverted compared to GI and LI; these differences may be due to low bacterial counts on L plates.

Sample 16 showed similar trend to sample 13, at day 2 for C and TS at the high AB concentrations (Figure 3(c)), but the initial counts with CI were 2 log-units higher than those from TSI plates. The initial psychrotrophic population had a high level of C-resistance (CI); a second “wave” of psychrotrophs, TS-resistant, was established after 48 h. As with sample 13, the counts on TS plates at day 4 were higher than those from C, suggesting that TS- and C-resistant psychrotrophs may not be the same isolates over time. If after 2 days of storage at 4°C bacterial isolates were resistant at levels of approximately 3, 3.5, and 4 log-units (of a total of 4.5) to G, C, or TS, respectively (for the lower AB concentration II, e.g., Figure 3(d)), we would assume that most had multiresistant features.

Together, the lower prevalence of TS-resistance compared to C-resistance at day 0 and the higher prevalence of TS-resistance than C-resistance at day 4 suggest that a first wave of psychrotrophs which is more C-resistant is replaced by a second population that is more TS-resistant. The second wave also frequently had G-resistant features; the resistance was after 2-day storage for 5 of 6 samples, about 2–3.5 log units lower compared to the corresponding controls. For most of these samples, no isolates grew on G-containing plates at day 0 or were present at low numbers (1 out of 100). Figures 3(a)–3(d) suggest a succession of psychrotrophic communities distinguishable by the prevalence of AR to the four ABs (representatives of four different AB classes) considered in this study, although a detailed analysis of the kinetics of the psychrotrophs that expressed AR features revealed a sample-dependent dynamic.

#### 3.2.4. Evaluation of AR for Raw Milk Samples Stored at 4°C and 6°C for 4 Days

**Descriptive Statistics**

The effect of cold storage on the AR of psychrotrophs (P) and mesophiles (M) was investigated at 4°C and 6°C for samples 1–6 and 7–12, respectively (Table 1). The “total counts” and psychrotrophs, enumerated on Mueller-Hinton plates without antibiotics, from samples stored at 6°C for 4 d, exceeded initial counts by 3.5–4 log-unit, and 4-5 log-units for the mesophiles and psychrotrophs, respectively (Table 1). Increases of 2–3.5 log-unit, and 2-3 log-unit for the mesophiles and psychrotrophs, respectively, were recorded when milk samples were stored at 4°C for 4 days, with the exception of sample 4 in which both populations types increased by approximately 1.5 log-units (Table 1). Samples 7–12 (Table 1) which were stored at 6°C exhibited the highest resistance at day 0 to ceftazidim (C): the AR levels ranged between 0 (sample 12) and 100% (sample 8) for both CIP and CIIP. With the exception of sample 8, in which psychrotrophs exhibited the highest AR to C, L, and TS, the AR for the mesophiles and psychrotrophs was low. After 4 days of storage, the AR generally decreased for all samples.

Samples 1–6 (Table 1), which were stored at 4°C, had the highest initial resistance levels to CII (31.4%–50% CIIM; 7.3%–56.7% CIIP) and to CI (20.5%–38.2% CIM; 7.3%–100% CIP) but also had higher AR to the other ABs in samples 1 and 2 (e.g., 27.2% for GIIP and 56.1% for TSIIP in sample 1) and to a lesser extent for samples 4 and 5.

**ANOVA Results**

The statistical analyses that distinguished the following factors (storage temperature of the milk, AB type, AB concentration, and bacterial population type) are detailed in Table 2. The determination of Rapd values as superior, inferior, or equal was based on the results of the mean comparisons of the Rapd values that were significantly superior, inferior or equal by the NPS tests. For the mesophiles (M), cold storage (4°C or 6°C) decreased the prevalence of AR to ceftazidim (C), regardless of storage temperatures, or AB concentration. Rap4 was lower than Rap0 for GI or GII at 4°C or 6°C, with the exception of GIM in which Rap4 was equal to Rap0. For trimethoprim-sulfamethoxazole (TS), Rap4 values were equal to Rap0, with the exception of TSIIM, where Rap4 was superior to Rap0. Although levofloxacin-resistant isolates were the most rare, Rap4 was equal to or greater than Rap0 when milk samples were stored at 4°C or 6°C, respectively.

The trends for gentamicin-resistant psychrotrophs (P) were similar at 4°C and 6°C (Table 2). Rap4 exceeded or was equal to Rap0 for GI and GII, respectively. Ceftazidim-resistance diminished after 4 days of storage at 4°C at both concentrations (CI, CII), but Rap4 values remained at the initial level in milk samples stored at 6°C. The observations with levofloxacin (L) and trimethoprim-sulfamethoxazole (TS) (Table 2) suggest that the milk storage temperature (4°C or 6°C) affected the AR trend, because at 6°C, all Rap4 exceeded the Rap0 values.

Bacterial growth is temperature dependent; even at low temperatures, a rise of 2°C enables the growth of more bacterial psychrotrophic species or genera [4] which was also verified in this study as “total counts” at 6°C were usually higher than those at 4°C (Table 1). Although, the study could not consider identical samples in both storage temperature conditions simultaneously, the AR trends obtained with four different ABs types (Table 2) suggest that the lowest storage temperature was most appropriate to contain AR at the minimal level.

#### 3.2.5. AR Evaluated at Days 0, 2, and 4 during Storage of Raw Milk at 4°C

**Descriptive Statistics**

**Figure 4:**
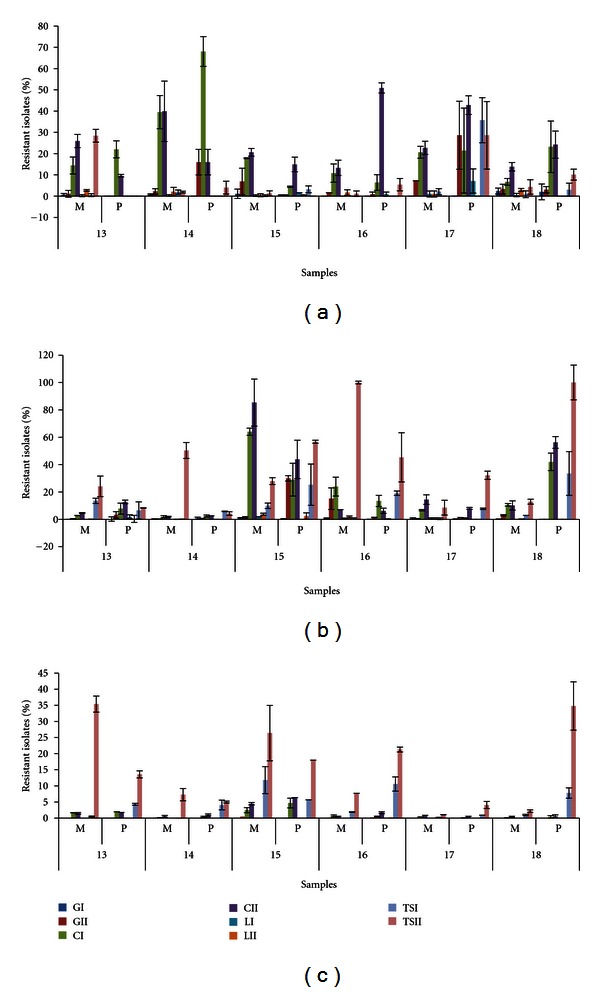
Percentages of resistant mesophiles (M) and psychrotrophs (P). Raw milk samples 13–18 were stored for 4 days at 4°C: day 0 (a), day 2 (b), and day 4 (c).

The counts from samples 13–18 at day 2 (Table 1) revealed a moderate increase compared with the initial bacterial load; at day 4, all counts exceeded 6 log-units on Mueller Hinton plates. At day 0, the AR prevalence was highest for ceftazidim whether considering the mesophiles (6.7%–39.5% and 13.8%–40% for CIM and CIIM, resp.) or psychrotrophs (4.4%–68% and 9.6%–50.9% for CIP and CIIP, resp.) (Figure 4(a)). Samples 14 and 17 had an AR prevalence of 16% and 28.6%, respectively, to gentamicin considering the psychrotrophs (GIIP). The highest AR prevalence for trimethoprim-sulfamethoxazole (TS) was observed for psychrotrophs in sample 17 and for mesophiles in sample 13. The lowest AR prevalence recorded was for levofloxacin (L) in both populations types (Figure 4(a)). Altogether the highest percentages of resistance were observed at day 2, and a sample-dependent shift was noticeable between days 0 and 2 (Figure 4(b)). An increase of AR was observed for sample 15 between days 0 and 2 for both mesophiles and psychrotrophs, whereas the trend was opposite in sample 17. Mesophiles from sample 16 and psychrotrophs from sample 18 exhibited up to 100% TS-resistance (Figure 4(b)). In terms of “total counts”, day 2 was an important sampling time point because the threshold level of 10^5^ CFU/mL was crossed in many samples.

At day 4, resistance to TS was the most prevalent, followed by resistance to C. The G- and L-resistant isolates were more rare but present at similar levels for both mesophiles and psychrotrophs (Figure 4(c)). When every AB is examined individually, the observation was made in a previous study that TS-resistance was more prevalent among isolates that spent a longer time in cold storage conditions, which was also noticed to some extent for some *β*-lactams antibiotics [25].

**ANOVA Results**

The statistical analyses revealed the same ranking of the four ABs for the mesophiles or the psychrotrophs regardless of the sampling day. C-resistance was the most prevalent at day 0, followed by TS, G, and L. At days 2 and 4, TS supplanted C without affecting the order of the two other ABs. The AR estimated through Rap0, Rap2, and Rap4 values for both types of populations was always the highest for psychrotrophs (Table 3). The range of variation of the Rap values, which were higher for psychrotrophs, confirmed higher heterogeneity among psychrotrophic communities. Most importantly, all Rap values were at their maximum for mesophiles and psychrotrophic populations at day 2, which corresponded to 48 hours storage at 4°C (Table 3).

Finnish raw milk is considered as excellent, with regard to both microbial loads and AB residues [30, 31]. The AR levels reported here and the trend of AR during cold storage raise the questions regarding the origin of AR and multiple AR in raw milk psychrotrophs. A direct link between the frequency of AB use and AR, which has been described worldwide, may be part of the explanation. In Finland, the prudent use of antimicrobials recommends that streptococci and *β*-lactamase-negative-staphylococci causing mastitis should be treated with systemically administered benzyl penicillin (*β*-lactams) [32] or intramammarily administered *β*-lactams combined with aminoglycosides [33]. The use of 3rd and 4th generation cephalosporins (like ceftazidim) in cattle is not permitted [32]. Acute clinical mastitis caused by Gram-negative bacteria may be treated systemically with trimethoprim-sulphonamide or enrofloxacin (fluoroquinolone) and severe coliform mastitis cases (approximately 12% of the cases in Finland) are treated with fluoroquinolones [32, 33]. 

Considering the AB classes considered here (ceftazidim-*β*-lactams, gentamicin-aminoglycosides, levofloxacin-quinolones, trimethoprim-sulfamethoxazole-folate pathway inhibitors), the differences in AR prevalences somehow reflect the AB usage frequencies with the exception of ceftazidim which is a third-generation cephalosporin for which coselection by other antimicrobials seems to influence resistance prevalence [34]. Although many agents can cause bovine mastitis (mammary gland infection), pathogens that induce clinical symptoms are mainly Gram-positive bacteria [35], whereas psychrotrophs are widely distributed among Gram-negative and less so among Gram-positive species [6]. Several studies that investigated the spoilage potential of raw milk psychrotrophs indicated that at the time of the spoilage, Gram-negative bacteria dominate the microflora [2]; however, the Gram status does not limit the transfer of AR traits [36, 37]. Since, apart from the cold storage, no other stress was applied to raw milk samples, the dominant factor that conducts the successional dynamics of the bacterial communities was resource availability over time. Considering the AR prevalence (Figures 3 and 4), three categories of bacteria could be roughly distinguished, as follows. (1) The first stage starts from the milking step and lasts until the dominance of psychrotrophs, in which the bacterial diversity (considering the numerous contamination sources) could be rather high. These bacteria are not yet so well adapted to cold storage, and nutrients are limited. The AR may be variable but not negligible. This stage could correspond to the domination of oligotrophs [38], depicted in Figure 3 (day 0) and Figure 4(a). (2) In the second stage, a pioneering group of psychrotrophs that produce exoenzymes (proteases, lipases, phospholipases) that degrade milk components (fats, proteins, carbohydrates, etc.) are dominant. This stage could constitute a transitory step between oligotrophs and copiotrophs [38, 39] and could correspond to communities plotted in Figure 4(b), for which the AR was highest. (3) In the third stage, four days of cold storage selected for authentic psychrotrophs that grow in a “richer” media and could be psychrotrophic copiotrophs (Figure 4(c)). AR is at its minimum in terms of relative amounts, which could suggest that carrying AR features may not be advantageous anymore. This hypothesis would be in line with the thought that bacteria produce ABs to kill or inhibit neighbouring bacteria when nutrients are limited [40, 41]. Considering that antimicrobial resistance mechanisms are associated with a fitness cost (which noticeably affects the bacterial growth rate) [42], we assume that the psychrotrophic enzyme producers that dominated at day 2 (Figure 4(b)) are outcompeted by a following psychrotrophic wave established at the cost of the enzyme producers. This wave multiplies quickly and carries fewer AR features (Figure 4(c)). 

Considering the AR load, milk at day 4 could have a lower risk (lower AR percentages) though this milk is spoiled and inappropriate for consumption. Noteworthy Figures 4(a)–4(c) indicate a considerable drop of the relative AR rates while the number of isolates that carry AR features continuously increased (Figures 3(a)–3(d)). 

Many genera constitute the rather highly diverse psychrotrophic raw milk microflora that enters the milk via numerous sources of contaminations [2, 5]; if many are considered benign and only regarded as spoiling agents, the presence of others, such as *Stenotrophomonas* or *Acinetobacter, *should be seriously considered due to their innate resistance to many ABs. Bacteria resistant to multiple ABs, known as superbugs, constitute one of the most challenging problems faced by modern medicine. One class is comprised of opportunistic pathogens, often of environmental origin that infect sick and immunocompromised patients [10, 14]. Still, little is known about the antibiotic resistomes of the majority of environmental bacteria [43]. 

This study revealed that no direct correlation can be made between CFU/mL values and AR prevalence. This addresses the question of whether the 10^5^ CFU/mL threshold “standard” is sufficient to ensure the quality and safety of raw milk. Indeed, heat treatments (pasteurization, UHT, etc.), to which raw milk is subjected, kill most or all bacteria; however, the results presented in this communication raise the question about the fate of all ARs genes loaded in mesophiles and psychrotrophs that may be released following heat treatment of raw milk. 

Some psychrotrophs isolated from 4 days-stored milk were subjected to rapid phenotypic characterization. According to the API 20NE database, some of the multiresistant isolates belonged to the species *Sphingomonas paucimobilis*, *Sphingobacterium spiritivorum*, *Chryseobacterium meningosepticum*, and *Stenotrophomonas maltophilia* (data not shown). Additional studies are necessary to accurately identify the bacterial species or genera that constitute the different communities and determine the link if any between AR load and ecological attributes (appartenance to copiotrophic or oligotrophic communities). The diversity of the different bacterial populations and their dynamics should be investigated by both culture-dependent and molecular methods. 

AR trends determined from the farm until the dairy silo would enable further evaluation of the present observations, further explanation on the impact of the cold chain on AR, and discussion of eventual connection between animal and human health. Strategies to limit the bacterial load of mesophiles and psychrotrophs in raw milk should be designed to dispel technological risks and simultaneously consider human health aspects. The cold chain of raw milk storage and transportation may be still seen as an amplification of benign bacteria, in their majority; however, the interplay between many benign and some risky bacteria should not be overlooked in a world that struggles with superbugs. 

## 4. Conclusion

The storage temperature and the antibiotic type influenced the AR prevalence, despite sample variations. During cold storage, different psychrotrophic communities that carry different AR levels seem to succeed to each other over time. When evaluated from relative amounts whether for psychrotrophs or mesophiles, the AR was most prevalent transiently at the investigated intermediate sampling point (48 h storage), at a stage where “total” counts are below or around 10^5^ CFU/mL where the milk is still acceptable for industrial dairy processes. 

## Figures and Tables

**Table 1:** “Total counts” ^§^on Mueller-Hinton agar and *R* values *.

Experiments	I				II				III			
Temperature	4°C				6°C				4°C			

Samples	log CFU/mL		*R* ^ a^		log CFU/mL		*R *		log CFU/mL			*R*
	Day 0	Day 4			Day 0	Day 4			Day 0	Day 2	Day 4	

**1**	5.0 ± 0.17	8.5 ± 0.17	1.01	**7**	4.0 ± 0.06	7.6 ± 0.10	0.80	** 13**	4.3 ± 0.08	5.5 ± 0.26	6.8 ± 0.09	1.00
**2**	3.7 ± 0.12	6.4 ± 0.02	0.94	**8**	4.0 ± 0.06	7.7 ± 0.02	0.76	** 14**	4.1 ± 0.16	5.3 ± 0.05	6.6 ± 0.16	0.76
**3**	3.2 ± 0.16	6.7 ± 0.16	1.09	**9**	3.6 ± 0.15	7.6 ± 0.03	0.77	** 15**	4.4 ± 0.04	4.4 ± 0.28	6.2 ± 0.08	0.93
**4**	4.0 ± 0.11	5.3 ± 0.12	0.79	** 10**	4.2 ± 0.09	7.9 ± 0.06	0.90	** 16**	4.1 ± 0.06	4.5 ± 0.21	6.7 ± 0.07	0.90
**5**	3.7 ± 0.01	5.8 ± 0.17	0.92	** 11**	4.2 ± 0.06	7.7 ± 0.04	0.92	** 17**	3.7 ± 0.10	4.4 ± 0.08	6.6 ± 0.14	0.76
**6**	3.9 ± 0.16	6.1 ± 0.21	0.82	** 12**	4.2 ± 0.10	7.9 ± 0.02	0.77	** 18**	4.0 ± 0.04	4.5 ± 0.26	6.7 ± 0.11	0.92

^§^Bacteria were enumerated after 3 days incubation at 30°C.

*The *R* value corresponds to the number of psychrotrophs divided by the number of mesophiles retrieved at day 0.

**Table 2:** Trend of Rap4 compared to Rap0 determined for raw milk samples 1–12.

Milk storage temperature	AB	AB concentration	Population type	Rap4 compared to Rap0^§^
4°C		I	M	Rap4 < Rap0
			P	Rap4 > Rap0
	G	II	M	Rap4 < Rap0
			P	Rap4 = Rap0
6°C		I	M	Rap4 = Rap0
			P	Rap4 > Rap0
		II	M	Rap4 < Rap0
			P	Rap4 = Rap0

4°C		I	M	Rap4 < Rap0
			P	Rap4 < Rap0
	C	II	M	Rap4 < Rap0
			P	Rap4 < Rap0
6°C		I	M	Rap4 < Rap0
			P	Rap4 = Rap0
		II	M	Rap4 < Rap0
			P	Rap4 = Rap0

4°C		I	M	Rap4 = Rap0
			P	Rap4 = Rap0
	L	II	M	Rap4 = Rap0
			P	Rap4 = Rap0
6°C		I	M	Rap4 > Rap0
			P	Rap4 > Rap0
		II	M	Rap4 > Rap0
			P	Rap4 > Rap0

4°C		I	M	Rap4 = Rap0
			P	Rap4 = Rap0
	TS	II	M	Rap4 > Rap0
			P	Rap4 = Rap0
6°C		I	M	Rap4 = Rap0
			P	Rap4 > Rap0
		II	M	Rap4 = Rap0
			P	Rap4 > Rap0

^§^The results correspond to the comparisons of the means of the Rapd values, from sampling days 0 and 4, by the NPS tests which are significant at the 0.01 level (for all tests performed).

**Table 3:** Mean values of Rapd (d = 0, 2, 4) and their corresponding range^§^.

	Mesophiles			Psychrotrophs		
Rapd	Rap0	Rap2	Rap4	Rap0	Rap2	Rap4

Mean	0.0788	0.2186	0.02402	0.1387	0.3336	0.0439
Range	0–1.1368	0–5.2363	0–0.5741	0–3.0000	0–9.0333	0–2.000

^§^determined for samples 13–18.

## Abbreviations

AB: Antibiotic
AR: Antibiotic resistance
G: Gentamicin
C: Ceftazidim
L: Levofloxacin
TS: Trimethoprim-sulfamethoxazole.
